# A Performance Management System in Healthcare for All Seasons?

**DOI:** 10.3390/ijerph17155590

**Published:** 2020-08-03

**Authors:** Milena Vainieri, Guido Noto, Francesca Ferre, Laura C. Rosella

**Affiliations:** 1 Management and Health laboratory, Institute of Management, Department Embeds, Sant’Anna School of Advanced Studies, 56127 Pisa, Italy; francesca.ferre@santannapisa.it; 2 Department of Economics, University of Messina, 98122 Messina, Italy; gnoto@unime.it; 3 Dalla Lana School of Public Health, University of Toronto, Toronto, ON M5T 3M7, Canada; laura.rosella@utoronto.ca

**Keywords:** performance management system, population, value, resilience, sustainability, healthcare

## Abstract

Health systems face challenges which are inherent to care demand and supply evolution (i.e., demographic change, new technologies) or are the results of unexpected occurrence originating outside the health system, such as economic shocks or epidemic outbreaks. Both challenges often require a paradigm shift in governance and organization, financing and resource allocation, accountability frameworks, as well as public health system responses. Based on key reviews and seminal papers of performance management, public health, sustainability and resilience, the article presents three emerging challenges for performance management systems in healthcare: i) the inclusion of the population approach; ii) the measurement and consideration of the multi-facets concepts of value; iii) the importance of resilience and sustainability. Performance management systems need to evolve to cope with this changing scenario. The article sheds light on uncovered areas by performance management, and it proposes a research agenda for scholars of both performance management and health service research.

## 1. Introduction

In his seminal paper of 1991, Christopher Hood highlighted the fact that the introduction of new public management (NPM) [1] was initially seen as an “all-purpose garment”, claiming a general applicability to different sectors and different contexts irrespective of the political context. However, the empirical evidence showed that countries accommodated NPM in different ways [2]—they applied the doctrinal components to stress and the underlying values they referred to [1]. The healthcare sector was one of the first movers to introduce NPM themes, which include disaggregation, competition and performance-based incentives [3], or following Ferlie’s [4] categorization, managerialization, markets and quasi-markets mechanisms and performance measurement.

In the healthcare sector, the introduction of specific standards and measures of performance pushed the development of multidimensional performance measurement systems at different governmental levels: international organizations [5,6,7,8], as well as national [9,10] and local institutions [11,12]. Moreover, around the 1980s, many reforms of healthcare systems could be ascribed to the principles of the NPM movement [13]. In four decades of application and implementation of performance management systems (PMSs) in healthcare, several changes occurred as a result of the unintended consequences that emerged and the new ideas regarding performance in healthcare [14,15,16].

Indeed, the evolution of performance management in the health sector is closely related to the development of the concept of “performance in health” itself. Performance represents the ability of an entity to achieve one or multiple desired results with attention to both the quality of actions and the quality of achievements (i.e., sustainable results) [17]. Hence, new visions and definitions of performance in healthcare require that the control of the activities and resources employed by health systems to achieve those results (performance management) must adapt.

**Figure 1:**
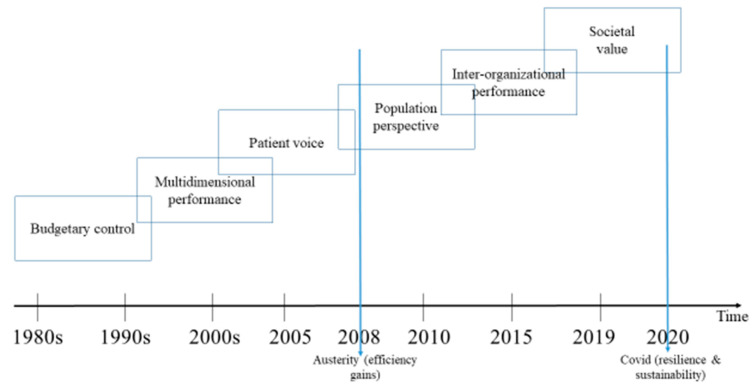
Development of performance management in healthcare: an overview of trends.

The evolution of the concept of performance in health is, in a way, a precursor of the changes in other sectors. Figure 1 summarizes the evolution of performance management in the healthcare sector as a consequence of the NPM reforms, which promoted the use of private-sector practices throughout the West [1].

The aim of NPM was to overcome the shortcomings of the traditional paradigm of bureaucratic public administration focused on efficiency and productivity intended as the objectives to be pursued by public organizations [1,18]. The performance management systems were designed and implemented in this early period in the health sector and thus were oriented at improving performance in terms of financial results, volumes of services provided—i.e., outputs and at identifying responsibilities within the organizational structure [19,20,21,22]. In the first two decades of performance measurement applications, more and more countries and umbrella organizations underlined the importance of measuring performance through multiple dimensions, also including users’ voices.

After 2000, a range of weaknesses and unintended consequences as a result of performance management arose in different sectors [18] that led to a new generation of performance management systems claiming to advance the complexity of the measures to be more comprehensive and support inter-organization performance as well as joint activities among different units in the same organization [23]. Similarly, numerous authors highlighted the continued pitfalls of measurement systems in healthcare [14,15]. In particular, the sub-optimization issue (i.e., focusing on improving one component of healthcare without considering the effects on the other components in the system) is particularly important both for the multilayer structure of the healthcare systems and the fact that often outcomes of care can be achieved through the strict collaboration of multiple entities and professionals—i.e., hospitals, general practitioners, department of prevention, etc. This requires the adoption of a concept of performance which goes beyond the outcomes achieved by single organizations or unit thus requiring to embrace the perspective of the population medicine [24,25] through the adoption of new tools and devices to foster the alignment of goals, exchange information, and promote joint actions between healthcare providers. Indeed, the population medicine approach has been considered as a paradigm shift in the way healthcare systems should behave.

Another critical change in performance management systems is the evolution of the concept of performance linked to value. Value in health has been defined by Porter [26] as the relationship between outcomes (namely results achieved in the delivery of a service) and costs (i.e., the resources employed to achieve those outcomes). This concept gained more and more relevance to the triple value framework developed by Berwick and its further adaptations [27,28,29]. As a consequence, these new concepts of performance were translated into new measurement and accountability tools.

Finally, emergency periods, such as the last fiscal crisis, and the recent COVID-19 epidemic, highlighted that performance should also embrace the concepts of sustainability and resilience. These unexpected occurrences also shed some shadows on the relationships between the central and local levels of governments, especially in decentralized healthcare systems [30].

We highlight only a few of the many changes that occurred in the “history” of performance management in healthcare over the past forty years. However, while there is a certain degree of agreement on the new perspectives of performance in health, these ideas have not been consistently embraced into the new garment of the performance management system in healthcare.

This article discusses the existing theoretical background related to value, population health, sustainability and resilience to build a fertile ground of discussion on what further research should explore in terms of strategies and tools (i.e., information systems) to advance PMS usefulness in health.

## 2. Aim of the Paper

Significant changes that occurred in healthcare in the last decades urge new considerations. This paper critically reflects and highlights uncovered areas to be further studied. We identify existing and emerging challenges that are pushing Performance Management Systems (PMS) to evolve. In doing this, we summarize the key studies of performance management, public health and resilience literature.

In particular, we base our analysis on (i) the established and well-known performance management frameworks in healthcare [5,11]; (ii) the seminal paper on the paradigm shift from standardized medicine to population and personalized medicine [24]; (iii) the new definition of value in healthcare [27], recently revised by the EU Expert panel group [29]; (iv) the sustainability and resilience framework [31]. From these streams of literature, we focus on the three main challenges that PMS has to consider: (i) population perspective, (ii) the multifaceted aspects of value and (iii) the potential trade-off between resilience and sustainability.

## 3. The Challenges of PMS to Capture Population Perspective

A population perspective encompasses whole populations (i.e., defined by geography, insurance coverage or attribution) and not only those with specific illnesses or needs. [32]. Misinterpretation of this concept is often at the root of failing to realize the true benefit of a population approach. For example, defining populations only by those that seek care or disease groups results in a fragmented and selected group that, when targeted, can leave others behind, widening inequities and reducing the benefits of disease prevention. A population health perspective recognizes that, beyond clinical services, social, economic and environmental factors greatly influence health and outcomes [33]. Furthermore, health outcomes are distributed among a continuum in populations and, in order to realize gains in the population, the full spectrum of needs must be addressed, not only the extremes of the distribution [34].

Historically, performance management has been focused on clinical processes and outcomes that apply to individuals and limited its ability to measure improvements to the health of populations [35]. There are several challenges to meaningfully capture a population perspective in a PMS that may explain the limited presence in current systems. These challenges relate to data availability, narrow concepts of measurement, multiple accountabilities over the broad determinants of health, and the lag time to observe prevention impact. The data challenges relate mostly to the relatively limited measures by which population risk is assessed. In order to characterize the full spectrum of risk from healthy to high-risk populations, consideration is needed for clinical and demographic, health behavior, patient preference and socioeconomic factors [36,37,38]. The Institute for Healthcare Improvement (IHI) emphasizes the broad range of metrics needed to capture a model of population health, including upstream factors, individual factors and a wide range of wellbeing, health and quality outcomes [39]. Existing population segmentation approaches purporting to support population health do not explicitly consider population factors, such as demographics, socioeconomic, and environmental factors [40]. Without measuring the factors and processes that influence population health, there is little hope in measuring value to the population. Beyond that, we must ensure the gains among subgroups in the population are at the expense of others, which results in widening health inequities and the worsening of the health of populations. The implementation of these ideas into PMS is limited in health systems around the world due to multiple sectors both within and outside of health being responsible for the upstream determinants of health. Given the broad account of the determinants of population health, PMS systems must adapt to be strongly integrated within regions where many levers for the determinants of health lie and measures strongly represent the range of population needs within. [41,42]. This includes integration with social systems [23].

One of the most significant challenges for PMS to capture population health is the importance of prevention in improving population health outcomes. Prevention approaches have the most significant potential to yield population health benefits yet take a long time to realize, which was signaled in Berwick’s original framing of the Triple Aim [27]. A successful population approach, with a strong focus on prevention, is also exemplified by “non-events”, such as keeping people out of the hospital or preventing disease. This makes measuring the impact more challenging. Overcoming these barriers is essential to ensure that the metrics reflect preventive activities that have the greatest potential for yielding population benefit. PMS will not be able to meaningfully support population health outcomes without more accurate definitions of populations, expansive measures of the processes and outcomes that determine health and prevent disease from a broad range of sectors and directly measure equity.

## 4. The Challenges of PMS to Capture Value Perspective

Current challenges for healthcare systems call for maximizing the value that healthcare organizations can provide to the population while ensuring a fair distribution of resources.

The concept of value in healthcare was first defined as the outcome achieved per dollar spent [26] following a price-based view of value. More broadly, value creation in the healthcare sector has been defined as that value that answers to the patient’s preferences (personal value), is based on quality standards (technical value), allows the correct allocation of resources (allocative value) [24,43] and considers the contribution of healthcare to social participation and connectedness (societal value) [29]. This broader and more comprehensive definition of value-based healthcare balances individual quality of care, patient experience, population health and wellbeing outcomes, with sustainability (financial, resource and environmental considerations) and equity considerations. Tensions between the different perspectives of value might emerge and prioritization is often based on the societal context (e.g., utilitarian vs. liberal society).

From this perspective, the core aim of healthcare systems is to maximize patient health benefits and to improve care experience through the design and implementation of integrated care pathways that ensure a cost-effective distribution of resources according to needs while also considering the equitable distribution of resources to different subgroups of the population. It is important to note that the value attached to health gains by the patients and by society can conflict—small health gains for an individual can be seen as highly valuable for the individual but may not translate to value for society. Where possible, both values should be captured, and where necessary, trade-offs be balanced to achieve allocative efficiency and prioritize interventions for socially deprived groups.

From this perspective, value assessment needs to be included in the systematic performance evaluation by considering both costs sustained and outcomes achieved during the care cycle experienced by patients from the diagnostic phase and therapeutic choices to the end-of-life [41,42] in a logic of high-value care (ensure that only interventions with strong evidence of cost-effectiveness are used). However, current performance management generally lacks comprehensive measures able to take into account the diverse values that come into play because they are also dependent on different stakeholder interests within a complex system. Traditionally, PMSs focus on measuring the process or output of a specific unit (provider) to reduce costs and increase efficiency and, more recently, minimize unwarranted variation in healthcare utilization [25]. However, to fully embrace the value concept, there is a need to consider what patients, healthcare providers, citizens and societies value most from their healthcare to also capture the interdependencies among the units and services along the care pathway.

An improvement in this direction is the inclusion and systematic evaluation of patient and caregiver perspectives among performance measurements to understand health gains/quality of life, preferences and overall care experience. There are recent examples where the perspective of the patient has been integrated into the performance systems both as a dimension of performance using indicators based on patient experience surveys [44], patient-reported outcomes [45], and as a mechanism to present performance information following the main phases of the patient care pathway [42]. Despite this increased focus, there are many challenges in successfully incorporating patient-reported information in a PMS that may explain the still limited use in current systems despite increased attention to the collection of patient-reported data for specific conditions or treatments to be used in clinical practice [46,47]. These challenges relate to data collection, response bias, implementing risk adjustment procedures [48], the appropriate time frame for data collection, data linkage and privacy issues, and the limited indicators with in-depth exploration using qualitative and more interactive tools (e.g., patient narratives).

The data challenges are not the only limitation in capturing the numerous value perspectives into performance systems. Indeed, there is need for a more broad reflection on the accountability for resources as a core aspect of professionalism by medical, nursing and other stakeholders stressing the importance of accountability for the health of the population, including the equitable distribution of resources for those with different diseases and those with the same conditions but poorer socioeconomic level. Additionally, long-term societally or collective goals (e.g., equity, social cohesion, population high-value care) and preferences (e.g., need assessment) are yet to be integrated into PMS mainly because of the difficulties in defying and measuring societal value itself [29].

A successful value-based approach, with a strong focus on patient and society, should, by definition, center on the valuation of benefits. However, we should avoid the risk of solely focusing on outcomes but instead create measures that consider the (opportunity) cost in resource allocation and complement such evaluation with traditional process and output indicators [49]. PMS will not evolve to meaningfully incorporate value-based healthcare without more refined measures of the outcomes (e.g., patients should be involved in measurement development), a shared definition of societal value that includes a commitment to universal health coverage—at least for NHSs [29], a correct balance between performance consideration at the patient and population level, and stronger clinical leadership to ensure the acceptance of responsibility for allocative efficiency and the societal impact of their decisions.

## 5. Future Challenges of PMS: Addressing Sustainability and Resilience of Health Systems

Sustainability and resilience are two “buzzwords” of the current decade. However, both in academic and political debate, we notice an improper use or conceptualization of these words.

Sustainability has been generally defined as the ability to meet “the needs of the present without compromising the ability of future generations to meet their own needs” [50]. It thus consists in guaranteeing that the multiple “resources” of a system are built and maintained over time through the management of their accumulation/depletion processes occurring at both the governance and individual level [31].

Focusing on health systems, we may identify three key resources [27,51]: the quality of care level, the equity level, and financial support. A sustainable health system operates by maintaining the desired level of quality, guaranteeing the same level of care response to every person (and over generations) and consuming only the financial resources allocated and not more. These three factors, which have been presented as separate elements, are strongly linked each other. For example, achieving and maintaining the desired level of quality of care requires adequate financial investment. On the other hand, quality of care means avoiding the emergence of costs related to mistakes, re-admissions, and so on. The same reasoning applies to the equity dimension. In fact, not guaranteeing equitable access and quality of care may provoke, at the population level, the emergence of health and social crises, the spreading of the disease to the “cared” population and epidemics, etc. This non-exhaustive and simplified set of examples demonstrates the difficulty in operating in a complex system in which interconnection between sub-systems and between the non-healthcare systems and the surrounding institutional environment are challenging to understand and assess fully. This also implies the need to engage and mobilize other forces outside the health system, which carries power and interests for health system functioning and, consequently, adds institutional complexity and fragmentation to its governance. Current PMSs lack the complete (or holistic) view of performance that would allow for the consideration of the aforementioned interdependencies and institutional fragmentation. Thus, future research into PMS in health should aim to support the development of solutions to cope with this challenge.

Resilience is another word that has disrupted our vocabulary in the last few years. Resilience can be defined as the ability to recover from or adjust to shocks [52,53,54], where shocks are defined as sudden and extreme changes which impact on health systems [55]. A resilient health system is thus able to resist and/or adapt to a natural or social event taking place. Resistance and adaptation support two characteristics that health systems need to be considered resilient: structural solidity (or preparedness) and flexibility (i.e., adaptive and transformative capacity). Structural solidity can be conceived as the endowment of resources which do not completely deplete after being exposed to a crisis or a change. This can be the case of a seasonal illness that produces an increase in healthcare demands. In this case, the health system should have sufficient capacity to rapidly absorb the increased demand and resulting service backlog (e.g., GPs visits). While structural solidity is certainly important to respond to small shocks or progressive changes, it cannot solely be considered enough when dealing with “black swans”—i.e., rare and unpredictable outlier events [56]. Nor can it be considered sustainable in a health system that accumulates and maintain resources to deal with extreme events during ordinary management. For example, during the peak of the COVID-19 pandemic in Italy, the use of intensive care beds was doubled. We cannot expect health systems to have twice the resources needed than in non-pandemic situations, since this would be seen as a waste of resources and undermine public value. Therefore, in these cases, health systems should rely on other characteristics that make them resilient while efficient, namely through flexibility. Flexibility allows for the alternative use of resources when dealing with a moment of crisis or change. A flexible health system can temporarily reallocate resources within a sub-system. Back to the COVID-19 example, flexible health systems may allocate beds, which are usually allocated for other types of care, such as for intensive care purposes. Another example could be related to the increasing prevalence of chronic disease; a flexible health system can reallocate resources among care-settings to provide an improved response to new care needs.

Lastly, in order to fully address resilience, a more rapid cycle of needs (including data production and management) and resources are needed to support decision-makers. The role of PMSs in this context is to support in assessing preparedness, shock management, and in building capacity for recovering and learning. To do that, PMSs are needed, among other challenges, to provide real-time evidence to effectively support decision-makers in assessing structural solidity and in adopting a flexible management style.

## 6. Discussion and Conclusions

The article provides an overview of the evolution in the design and use of performance management in healthcare and presents current challenges. In particular, we focused on the current challenges in performance management that are needed to cope with the increased complexity, interdependencies and unplanned experiences in contemporary health systems. We believe that these challenges may be addressed by focusing on the evolution of the concept of performance itself. Accordingly, we identified three areas that are guiding scholars toward the evolution of theories and practices related to performance management in healthcare, namely: value, population, sustainability and resilience.

Although presented in three separate sections, these concepts are highly intertwined at a substantial level. For example, if creating value means to maximize the outcomes through the deployment of the given resources, the population perspective supports the adoption of a broader view answering to the question “value for whom?”. At the same time, sustainability and resilience support the responsible use of what was previously called “given resources”, to maintain them for future generations resisting and also reacting to the emergence of potential external shocks.

The adoption of a systemic (or holistic) framework for performance measurement is supported by recent scholarly contributions in this area. Such a holistic view implies the understanding of the internal and external environment in which healthcare is performed, and recognizing the integrated and concurrent nature of these challenges.

In the population perspective, the holistic view is mainly intended as the ability of the healthcare sector to take into account how the delivery of healthcare services directly and indirectly impacts on the people receiving care, on those that do not receive it and on the society in general.

The value-based perspective also requires the adoption of a holistic view, meaning a less individually-focused approach (patient-centered), as initially claimed by Porter [26], and a more socially inclusive perspective that considers, for instance, equity aspects.

From a sustainability and resilience perspective, the holistic view is mainly intended as the ability to guarantee that resources are used and built to preserve them for future generations and responsibly allocated public investment.

In summary, we confirm that a systematic approach in performance management is needed to overcome a static representation of health systems both in terms of the components—i.e., to avoid that performance is conceived as the performance of a professional or a unit for a specific patient—and time—i.e., to prevent myopia in the delivery of healthcare services only on short-term impacts.

What should performance management in healthcare focus on in order to support the challenges of the adoption of such a holistic approach and its related challenges? Many topics could be further explored to contribute to this.

First of all, improvements in data collection management and use are essential. Today, big data and artificial intelligence are two areas that show great potential to support the design and implementation of PMSs through the rapid integration of more complex data streams. These may foster the ability to get real-time information and to improve the forecasting capacity of organizations and the design of more customized responses to patient and society needs.

Second, tools to adopt a patient-based perspective are welcome to improve the ability of professionals, units and providers to be more patient-centered together with improved ability to also capture population performance.

Third, PMS in healthcare organizations should be designed to support the link between their operations (i.e., how services are delivered) and the strategy at the organization and health system level.

Lastly, how performance measures are used in practice requires a particular focus (i.e., implementation). Further, fostering shared responsibility on the results achieved between different healthcare and non-healthcare institutions may allow for overcoming the “silos” mentality for which only results within one specific area are reported and acted upon and to instead focus on the creation of value across the system and for the population.

In this contribution, we identified performance-measurement challenges of the future, thus presenting the community with an opportunity for developing research in the response to these challenges.
